# Investigating linkages between human movement and meteorological variables on dengue outbreaks in the Pacific Islands

**DOI:** 10.1371/journal.pntd.0013607

**Published:** 2025-10-22

**Authors:** Justin Sexton, Tanya Russell, Thomas R. Burkot, Adam Craig, Roslyn I. Hickson

**Affiliations:** 1 Commonwealth Scientific and Industrial Research Organisation (CSIRO), Townsville, Queensland, Australia; 2 Australian Institute of Tropical Health and Medicine, James Cook University, Townsville, Queensland, Australia; 3 Centre for Clinical Research, University of Queensland, St. Lucia, Queensland, Australia; Universite de Montreal, CANADA

## Abstract

The Pacific Island Countries and territories (PICs) experienced a doubling of annual reported dengue outbreaks between 2012 to 2019, including concurrent outbreaks of multiple dengue serotypes. This has major health implications for the region as reinfection can lead to more serious health complications. Decision support systems for dengue can mitigate the risk of outbreaks by providing information on which early planning and proactive interventions may be based. Such decision support systems require an understanding of the factors that drive dengue outbreaks. Current efforts to build decision support tools, such as disease forecasting models, rely on links between environmental factors and dengue outbreaks, largely ignoring human movement. To address this gap we used random forest and XGBoost models to analyse potential links between human movement and meteorological variables on dengue outbreaks in PICs. We used variable importance metrics and a forward selection process to identify key combinations of explanatory variables. The findings highlighted that the two-month lead average minimum temperature was an important indicator of both months when an outbreak was current (“outbreak month”) and the month of the start of outbreaks (“start month”). In comparison, international arrivals from outside the Pacific Islands was only considered important for the start month. These results were consistent whether random forest or XGBoost was used to build classifier models. Despite some differences in variables selected, forward selection resulted in similar performance for both random forest and XGBoost models. The models developed in this study were exploratory and require further development before use as a policy tool. Future research into dengue risk in PICs should further explore the impact of human mobility between countries on dengue outbreaks.

## Author summary

Increasing outbreaks of dengue are a major health concern for Pacific Island Countries and territories (PICs). Understanding the risk factors associated with increases in transmission and the occurrences of dengue outbreaks is critical for supporting improved preparedness and outbreak responses. We used two tree-based classification models and a forward selection procedure to investigate links between human movement and meteorological variables on dengue outbreaks in PICs. Using this approach, we identified minimum temperature and global human movement as consistent explanatory variables of the start month of an outbreak across both models, which had similar performances. Our results highlight the need to further investigate the role of human movement when developing outbreak forecasts or decision support tools.

Fri Oct 17 15:38:51 2025

## Introduction

**Fig 1:**
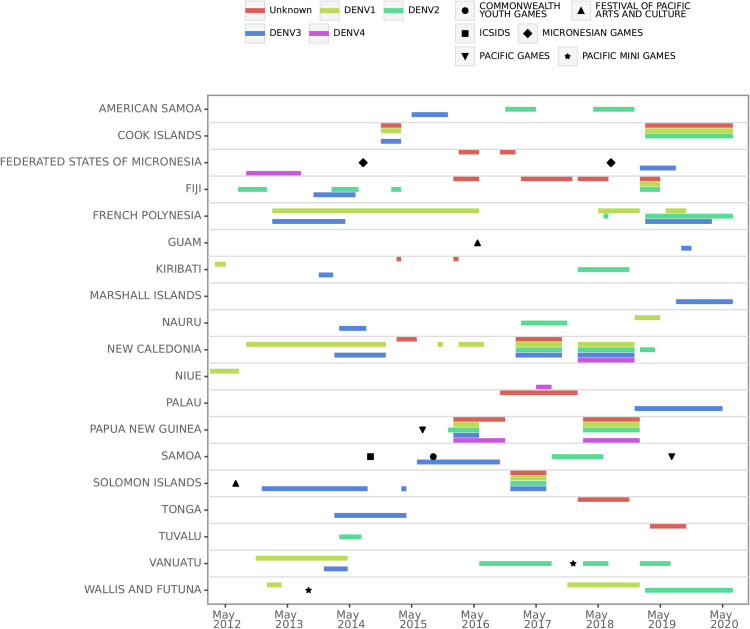
Outbreaks and international mass gatherings in the Pacific Island Countries 2012–2020. The outbreaks are based on dengue-like-illness surveillance and are depicted as bars showing the start date and duration. The six categories of mass gatherings are identified as the 10 points on the timeline.

Dengue fever is a growing global tropical public health concern, with a 10-fold increase in suspected cases over the last 20 years [1]. Annual reports of dengue outbreaks in Pacific Island Countries and territories (PICs) doubled from 2012 to 2019, with an increase in multi-serotype outbreaks Fig 1, which have serious and even deadly consequences. More recently, reported cases of dengue like illness increased by 28% from 2022 to 2023 [1]. Research into the factors that lead to outbreaks is essential to enhance the preparedness and response to control dengue outbreaks in the PICs. However, developing these measures requires understanding the conditions associated with dengue outbreaks.

Dengue fever is caused by the dengue flavivirus and is transmitted to humans by *Stegomyia* mosquitoes in the *Aedes* genus. Twelve mosquito species are competent dengue vectors in PICs, with *Aedes aegypti* and *Aedes albopictus* being primary vectors [2]. As the presence of vectors is fundamental to dengue transmission, most research into dengue outbreaks have considered the link between meteorological variables and *Aedes aegypti* or *Aedes albopictus* populations [3–7]. These researchers found that temperature, rainfall and humidity influence *Aedes* populations and dengue outbreaks.

Meteorological variables can be predictors of dengue occurrences and outbreaks [3,4,8,9]. Models incorporating human movement into research on understanding dengue outbreaks is still sparse [10,11], with 70% of publications in a recent review considering only meteorological variables [8], despite evidence that human movement is a driver of dengue spread [12–17].

Some recent dengue forecasting has considered human movement both mechanistically [18–21] and using machine learning approaches [10,11,19,22,23] with most studies using mobile phone or social media data as a surrogate for human mobility. The high spatial and temporal resolution provided by such data facilitates analyses of dengue dispersal within but not between countries. Many statistical and machine learning models have been used to model dengue outbreaks [8,9].

Random forest [24] is a tree-based ensemble learning technique with increased ability to correctly forecast dengue case numbers when compared with other modelling techniques [25–29]. Random forest models are built by creating a large number of decision trees, with each tree built on a sample of the data. Extreme gradient boosting (XGBoost; [30]) has also been used widely in predicting dengue outbreaks [25,31,32]. Similar to random forests, XGBoost is a tree based method. XGBoost expands on gradient boosted tree-based methods, including regularization and shrinkage that can help prevent model overfitting [30,31]. XGBoost and Random Forest have shown to be comparable in model performance and preform as well as or better than other modelling approaches tested [25,31].

An advantage of tree-based modelling is the capability of understanding how influential a variable is within the model (that is, variable importance). This variable importance can be used in an important stage of model development referred to as “variable selection” to simplify and improve the model performance by removing redundant variables. Although some research has leveraged this variable importance property of random forest models for dengue forecasting models [27,33], they have not used a forward selection approach, which has been shown to improve random forest-based predictions for other applications [34,35]. Forward selection is a variable selection approach that iteratively adds the variable that most improves model performance (e.g. accuracy) until performance stops increasing.

Arbovirus transmission in small island nations can have differing patterns to large continental areas. As many PICs are small with human mobility between them primarily by air, case studies of the PIC dengue outbreaks offer a unique opportunity to identify the contribution of human movement to dengue outbreaks. While some islands are large enough to sustain endemic dengue transmission (such as Fiji, Papua New Guinea, Solomon Islands and Vanuatu), many smaller nations often see a pattern of large outbreaks that burn out when the population reaches a seropositive threshold. Followed by a period with minimal transmission during which the seropositivity of the population declines. When outbreaks do occur, they often overwhelm the already fragile health system. Identifying potential drivers and explanatory variables of outbreaks could lead to the more proactive implementation of early intervention strategies to mitigate outbreak risk.

We built descriptive models to explore competing understandings of whether meteorological variables or human movement have historically been important for dengue outbreaks in PICs. To achieve this, we built random forest models to classify the “start month” (the month in which the outbreak was reported to start) of an outbreak or “outbreak months” (months in which a country was considered to have a current outbreak) based on reports of dengue-like-illness in PICs. We then explore the best combinations of explanatory variables to explain the historic data.

## Materials and methods

Data on dengue outbreak between 2012 and 2020 [36,37] were used to investigate the importance of international mass gatherings and human mobility on the emergence of dengue outbreaks. By human movement we refer to international flight arrivals into a country from other PICs (referred to as regional travel) or from any other country in the world (referred to as global travel) or international mass gathering events (referred to as mass gatherings throughout). Data on international global and regional (within PIC) flights and international mass gatherings were collected and collated to a monthly timescale from 2012 to 2020. Random forest and XGBoost models were then used to classify month-country combinations as start months and outbreak months using monthly passenger data and mass gathering occurrence indices as potential explanatory variables. The average correct classification rate across classes (balanced accuracy) was compared when different combinations of variables were used and variable importance measures were explored to identify influential variables. Finally, a forward selection process was used to rebuild models and identify the best combination of variables for each model.

### Data

#### Study region.

For the purposes of this paper, we define Pacific Island Countries and territories (PICs) as 19 of the 27 island countries and territories located in the North and South Pacific Ocean, that are Member States of the Pacific Community [38] and that were reported to have experienced at least one dengue outbreak during the study period (see Sect Dengue outbreaks). These PICs are: American Samoa, Cook Islands, Federated States of Micronesia, Fiji, French Polynesia, Guam, Kiribati, Republic of the Marshall Islands, Nauru, New Caledonia, Niue, Palau, Papua New Guinea, Samoa, Solomon Islands, Tonga, Tuvalu, Vanuatu, and Wallis and Futuna.

#### Dengue outbreaks.

Monthly start and end dates of dengue outbreaks affecting the 19 PICs were taken from Table 1 of [36] (2012–2014) and the Supplementary Table 1 of [37] (2014–2020). Months in which an outbreak was first recorded were defined as “start months” while “outbreak months” were defined as months in which an outbreak started or was ongoing. These definitions included outbreaks of any serotype. Outbreak months that had concurrent outbreaks of all four known dengue serotypes were removed from the analysis of outbreak start months.

**Table 1:** List of variables used in modelling and their descriptions.

Variable	Description
**Target Variables**	
start month	Binary variable identifying if an outbreak started in an observed month.
outbreak month	Binary variable identifying if an outbreak was current in an observed month.
**Human movement explanatory variables**	
international event(lag1,lag2)	A binary variable identifying if an international event occurred in an observed month, the month before (lag1) or two months before (lag2).
regional arrivals(lag1, lag2)	A numeric variable representing the detrended, mean centred and standard deviation scaled arrivals from within the PIC countries in an observed month, the month before (lag1) or two months before (lag2).
global arrivals(lag1, lag2)	A numeric variable representing the detrended, mean centred and standard deviation scaled arrivals from countries other than PIC countries in an observed month, the month before (lag1) or two months before (lag2).
regional arrival event(lag1,lag2)	A binary variable identifying if regional arrivals were high (above the 75th Percentile) in an observed month, the month before (lag1) or two months before (lag2).
global arrival event(lag1, lag2)	A binary variable identifying if global arrivals were high (above the 75th Percentile) in an observed month, the month before (lag1) or two months before (lag2).
**Meteorological explanatory variables**	
average max temp(lag1, lag2)	average daily maximum temperature for each observed month, the month before (lag1) or 2 months before (lag2).
average min temp(lag1, lag2)	average daily minimum temperature for each observed month, the month before (lag1) or 2 months before (lag2).
total rain(lag1, lag2)	total rainfall in a month, last month (lag1) or 2 months ago (lag2).
total radn(lag1, lag2)	total radiation in a month, last month (lag1) or 2 months ago (lag2).
average max rh(lag1, lag2)	average daily maximum relative humidity for each observed month, the month before (lag1) or 2 months before (lag2).
average min rh(lag1, lag2)	average daily minimum relative humidity for each observed month, the month before (lag1) or 2 months before (lag2).

Three cases of overlapping outbreaks were merged:

- The outbreak of serotype 1 in French Polynesia that was still active in August 2014 in [36], was merged with the corresponding serotype 1 outbreak in [37].
- The outbreaks of serotype 1 and serotype 3 that were still active in New Caledonia in August 2014 in [36], were both merged with the ‘unknown’ serotype outbreak in [37].
- The outbreak of serotype 3 in Tonga that was still active in August 2014 in [36], was merged with the corresponding serotype 3 outbreak in [37].

Merging of secondary data provided a consolidated log of dengue outbreaks by serotype across the period 2012 to 2020. The final timeline of outbreaks and identified Pacific-based mass gatherings is shown in Fig 1.

#### Human mobility data.

We used OAG historical flight passenger arrival numbers into a country (https://www.oag.com/historical-flight-data) as our human mobility data. Passenger arrival and departure numbers were available at a monthly time step for each PIC from January 2012 to December 2020. Monthly global data on arrivals into PICs were subsequently summarised into two different categories: (i) “global international travel” (flights that arrived at a PIC on a flight that originated in a non-PIC country) and (ii) “regional international travel” which are defined as passengers that arrive at a PIC on a flight that originated in another PIC.

#### Identifying international mass gatherings.

Large scale international mass gatherings within the study period were identified based on records of events from the regional enhanced surveillance programme of the Pacific Community [39]. In total, ten events were identified during the study period (Fig 1): The Festival of Pacific Arts in 2012 (Solomon Islands) and 2016 (Guam); The Pacific Mini-Games in 2013 (Wallis and Futuna) and 2017 (Vanuatu); The Pacific Games in 2015 (Papua New Guinea) and 2019 (Samoa); The Commonwealth Youth Games in 2015 (Samoa); The Micronesian Games in 2014 and 2018 (Federated States of Micronesia); and The 3rd International Conference on Small Island Developing States in 2014 (Samoa). Note in Fig 1 there are ten events from six categories of international mass gatherings.

#### Approximating months with mass gatherings based on flight arrivals.

Given the small number of international mass gathering events identified in Sect Identifying international mass gatherings, two sets of flight-based mass gatherings variables were developed: regional flight events and global flight events. These two variables were defined as months with arrivals greater than the upper quartile (75th percentile) of all month-country combinations for arrival data from other PICs (regional flight events) and arrival data from any other country (global flight events). Using percentile thresholds of 75, 55 and 95 resulted in 447, 804 and 90 regional flight events, which respectively captured six, nine and three of the ten known events throughout the PICs. Based on these results, a threshold of the 75th percentile was chosen as a balance between capturing a reasonable portion of the identified mass gatherings (six), and suggesting a large number of mass gathering events across the PICs.

#### Meteorological data.

We represented the meteorological data for each country by the weather of the capital city. Meteorological data for 2011–2020 was sourced from Open-Meteo [40]. Hourly data were extracted using coordinates of capital cities in each PIC. This data uses ERA-5 and ERA5-Land re-interpolated data on a grid for the whole globe. Open-Meteo selects the closest grid point to the selected location. For some islands, land based estimates were not available and the closest ocean-based data were used. Hourly weather data estimated daily total rainfall and solar radiation as well as daily maximum and minimum humidity and temperature.

### Analysis

#### Data pre-processing.

Several steps were taken to process the data into explanatory variables.

Explanatory variables based on meteorological data were created at the monthly time step as either the average of daily values (maximum and minimum temperature, maximum and minimum relative humidity) or as monthly totals (rainfall, radiation).

Flight data were detrended for each country to remove both the increase over time and seasonal trends. This was achieved in Python version 3.11.0 [41] using seasonal trend decomposition from the *statsmodel* version 0.13.5 package [42]. Detrended flight data were also mean centred and standard deviation scaled using the *Scale* function from the *Scikit*–*Learn* version 1.5.2 python package [43]. Mass gatherings based on flight arrivals (see Sect Approximating months with mass gatherings based on flight arrivals) were based on these detrended data.

Each explanatory variable was recalculated at a one– and two–month lag (value from last month and value from two months ago, respectively) to capture potential offsets between a change in conditions and an outbreak being recorded. A complete list of variables and targets was recorded in Table 1.

#### Random forest modelling.

The random forest [24] algorithm was used to model outbreak start and outbreak months. Specifically, random forest models were built using the *randomForest* version 4.7-1.2 package [44] in the R version 4.4.0 statistical environment [45]. Three models were built for each target variable, a model using only human movement and mass gathering data, one using only meteorological data and one using meteorological, human mobility and mass gathering data Table 1.

The analysis was completed in three steps:

1. *Hyper-parameter tuning through five-fold cross-validation*. The maximum number of final nodes (*maxnodes*), number of trees (*ntree*) and number of variables trialled at each split (*mtry*) were tuned through five-fold cross-validation using a grid search technique. A range of 2,3,4,...,29 was used for *mtry* while a range of 500,550,600,...1000 was used for *ntree*. For *maxnodes* an initial range of 5,10,15,...,50 was used before being refined to 1,2,...,10 based on cross-validate performance in balanced accuracy. Mean and standard deviations were recorded for the cross-validated balanced accuracy, sensitivity and specificity. Balanced accuracy was calculated as the mean of sensitivity and specificity, where sensitivity was the true positive rate (correct classification rate of the positive class) and specificity was the true negative rate (correct classification rate of the negative class). For example, in modelling start months, sensitivity is the percentage of start months correctly classified as such while specificity is the percentage of non-start months correctly classified as non-start months.
  To account for imbalance in the target variable categories, minority classes (e.g. months in which an outbreak started) were randomly up-sampled with replacement. Up-sampling was performed in the calibration sets only during cross-validation. Results were recorded for the best set of hyper-parameters based on the cross-validated balanced accuracy. Results for the best tuned model were used to compare model performance and in all further analysis.
2. *Variable Importance.* In order to explore variable importance, each model was rebuilt on the entire dataset, using the hyper-parameters defined in the tuning stage. Up-sampling was again used to offset class imbalance. Variable Importance was recorded as the mean decrease in accuracy. Relative importance was calculated using a min-max scaling such that the most important variable had a relative importance of 1 while the least important had a relative importance of 0. As up-sampling was done with random replacement, models were rebuilt 1000 times and the mean and standard deviation of relative variable importance was recorded.
3. *Forward selection random forest.* Finally, to investigate explanatory variables that work well in combination, and to produce more parsimonious models, a forward selection algorithm (see S1 Algorithm) was used to rebuild the random forest models. Starting with the most important variable, random forest models were built and the five-fold cross-validated balanced accuracy was recorded. Models were then iteratively rebuilt by adding each variable in turn, and the variable that increased the balanced accuracy the most was added to the model until balanced accuracy no longer increased by more than 1%. Variables included in the final models were recorded along with final cross-validated balanced accuracy, sensitivity, specificity, precision and F1 score.

#### Gradient boosted trees.

The extreme gradient boosted regression tree approach (XGBoost [30]) was used for comparison to the random forest models. Similar to random forests, XGBoost is an ensemble method based on decision trees that attempts to iteratively improve predictions using information from previous trees. XGBoost was implemented using the R software package *xgboost* version 1.7.8.1 [46].

Analysis followed the steps outlined in Sect Random forest modelling. For hyper-parameter tuning through cross-validation, the maximum depth of trees (*maxdepth*), subsampling ratio(*subsample*), number of boosting iterations (*nrounds*), and step size shrinkage (*eta*) parameters were tuned using a grid search technique. The ranges used for the grid search were 2,4,...,10,15,20,...,50, 0.2,0.4,...,1, 2,4,...,20, and 0.2,0.4,...,1, respectively. Classification models were built as binary logistic regression, using boosted trees. Model performance metrics and feature importance were recorded.

Importance of variables in XGBoost models were based on the “Gain” importance measure calculated by the *xgboost* package. Variables that were not reported by *xgboost* were considered to have a value of zero. As with random forest modelling, a min-max scaling was used so that a relative importance value of one was given to the most important variable in any individual modelling run.

## Results

### Model comparison through cross-validation

We used five-fold cross-validation to optimise hyper-parameter selection when building both random forest and XGBoost models to classify start months or outbreak months. Models were built using human mobility, mass gathering and meteorological variables together; human mobility and mass gathering data only; and meteorological variables only. All potential models resulted in relatively low balanced accuracy in classifying either start months or outbreak months (Table 2).

**Table 2:** Model classification skill for cross-validation of hyper-parameters for random forest and XGBoost models. Definitions of variables are provided in Table 1.

Target	Explanatory variables included	Balanced accuracy (StdDev)	Sensitivity (StdDev)	Specificity (StdDev)	Hyper-parameters
**random forest**					
Start month	human mobility mass gatherings meteorological	59.0(4.9)	30.6(9.9)	87.3(9.9)	*ntree*: 700 *mtry*: 20 *maxnodes*: 7
	meteorological	56.9(3.9)	23.8(4.1)	90.1(4.1)	*ntree*: 800 *mtry*: 1 *maxnodes*: 10
	human mobility mass gatherings	55.7(3.1)	20.3(9.4)	91.1(9.4)	*ntree*: 550 *mtry*: 11 *maxnodes*: 3
Outbreak month	human mobility mass gatherings meteorological	58.9(7.3)	57.3(17.0)	60.6(17.0)	*ntree*: 650 *mtry*: 12 *maxnodes*: 6
	meteorological	59.7(7.2)	55.0(14.0)	64.4(14.0)	*ntree*: 600 *mtry*: 17 *maxnodes*: 9
	human mobility mass gatherings	55.3(4.3)	32.5(8.8)	78.1(8.8)	*ntree*: 700 *mtry*: 15 *maxnodes*: 9
XGBoost					
Start month	human mobility mass gatherings meteorological	60.2(7.6)	38.9(10.9)	81.6(4.3)	*eta*: 0.4 *maxdepth*: 2 *nrounds*: 4 *subsample*: 0.4
	meteorological	61.7(3.6)	37.3(7.2)	86.0(1.6)	*eta*: 1 *max*_*d*_*epth*: 15 *nrounds*: 2 *subsample*: 0.2
	human mobility mass gatherings	58.0(11.4)	42.7(21.6)	73.3(3.5)	*eta*: 1 *maxdepth*: 4 *nrounds*: 2 *subsample*: 0.2
Outbreak month	human mobility mass gatherings meteorological	59.1(3.6)	52.6(6.7)	65.7(1.4)	*eta*: 0.2 *maxdepth*: 2 *nrounds*: 10 *subsample*: 0.4
	meteorological	59.3(3.0)	45.6(3.4)	73.0(3.7)	*eta*: 0.2 *maxdepth*: 20 *nrounds*: 6 *subsample*: 0.4
	human mobility mass gatherings	53.5(1.9)	52.8(5.5)	54.2(5.6)	*eta*: 0.8 *maxdepth*: 4 *nrounds*: 4 *subsample*: 0.2

The maximum balanced accuracy achieved by random forest modelling was 59.0% and 59.7% (bolded values in Table 2) for classifying start month and outbreak months respectively. The maximum balanced accuracy for the start month was achieved by the model that used human mobility, mass gatherings and meteorological variables. The maximum balanced accuracy achieved for classifying outbreak months was achieved by the model that used only meteorological variables (Table 2). For both targets, the use of only human mobility and mass gatherings variables resulted in the lowest balanced accuracy.

Similarly, for XGBoost models, the lowest balanced accuracy occurred using only travel data. However, for both start month and outbreak month models, the highest balanced accuracy (bolded values in Table 2) were achieved using only meteorological data. For start month using only meteorological variables resulted in a mean balanced accuracy of 61.7% while outbreak month using only meteorological variables had a mean balanced accuracy of 59.3%. Balanced accuracy means were generally within one standard deviation of each other whether comparing between random forest and XGBoost or between variable sets using the same modelling approach. A notable exception was the XGBoost model for outbreak month using only human mobility and mass gathering events, which had the smallest mean balanced accuracy (53.5%) and a comparatively small standard deviation (1.9%).

The optimised hyper-parameter values are recorded in Table 2, which were then used in subsequent modelling. The hyper-parameter optimisation identified similar values for all random forest models. Hyper-parameters for XGBoost models varied. In particular, XGBoost models using only meteorological variables used much higher maximum depths (more complex trees) than other models.

### Model variable importance

**Fig 2:**
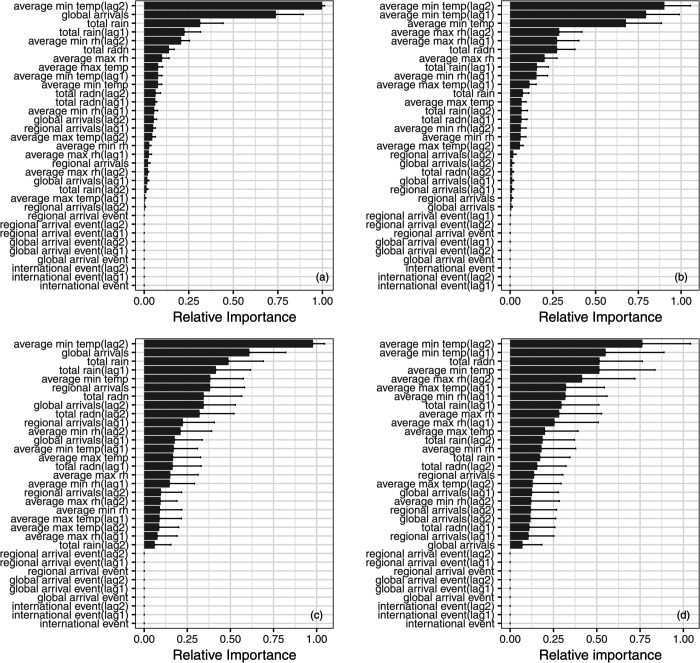
Relative variable importance for estimating the start month (a,c) and outbreak months (b,d) using random forest (a,b) and XGBoost (c,d) based on human mobility, mass gatherings and meteorological variables. Bars represent the average relative importance across 1000 random up-samples. Black lines represent one standard deviation.

The relative importance of variables within each model was explored using the optimised hyper-parameter settings. To capture modelling process uncertainty, the models were rebuilt 1000 times. For brevity, here we focus on showing differences in relative importance for outbreak start and outbreak month models, where all potential explanatory variables were included (Fig 2).

For random forest models classifying both start month (Fig 2A) and outbreak months (Fig 2B), the average minimum temperature two months prior (average min temp(lag2)) was generally considered the most important explanatory variable. For start month, the sequentially important variables were global travel (international arrivals from outside the PICs) in the current month (global arrivals) and total rainfall one month prior (total rain(lag1)). Regarding outbreak months, average minimum temperature at one month lag (average min temp(lag1)) and average minimum temperature of the current month (average min temp) were the second and third most important variables within the random forest model. For both start month and outbreak months, at least one relative humidity variable was included in the top five variables, while a solar radiation variable was included in the top ten.

Variable importance for the XGBoost models were similar to results from the random forest models (Fig 2C, 2D). For the start month, the top three variables were identical to the random forest model, while for the outbreak month model, the average minimum temperature variable was replaced by total radiation as the third most important variable. For the start month, the XGBoost model did not include a relative humidity variable in the top five, but two regional arrival variables were included in the top 10. Similarly to the random forest models.

For classifying both start month and outbreak month using either random forest or XGBoost, known mass gathering variables (international event, international event(lag1/lag2)), mass gathering variables based on arrivals from other PICs (regional arrival event, regional arrival event(lag1), regional arrival event(lag2)), and mass gathering variables based on arrivals from outside the PICs (global_event, global arrival event(lag1), global arrival event(lag2)) were not considered important in either random forest or XGBoost models.

### Exploration of model variable combinations through forward selection

To explore the combinations between different factors affecting dengue outbreaks, a forward selection algorithm was employed to develop models focusing on the most important variables identified earlier (see Fig 2). The results of the forward selection models, including their performance metrics, are summarised in Table 3.

**Table 3:** Model classification skill for cross-validated forward selection of the random forest and XGBoost models. Further descriptions of the variables used can be found in Table 1.

Target (included variables)	Balanced Accuracy (StdDev)	Sensitivity (StdDev)	Specificity (StdDev)	Precision (StdDev)	F1 score (StdDev)
**random forest**					
Start month (av min temp(lag2), total rain(lag1), global arrivals,regional arrivals)	64.2(7.0)	42.6(15.2)	85.8(3.3)	9.2(2.8)	0.151(0.048)
Outbreak month (average min temp(lag2),total radn)	59.7(3.3)	66.9(3.0)	52.5(5.2)	34.8(3.0)	0.457(0.031)
**XGBoost**					
Start month (av min temp(lag2), global arrivals)	60.5(4.5)	39.0(12.1)	82.0(3.7)	6.8(2.4)	0.112(0.035)
Outbreak month (average min temp(lag2), total radn)	59.5(2.9)	61.6(3.8)	57.4(3.6)	35.4(2.8)	0.449(0.031)

The use of forward selection resulted in more parsimonious models using either random forest or XGBoost. Mean balanced accuracy increased for the random forest start month model, but remained similar or decreased for all other models. Sensitivity tended to increase when forward selection was used, while specificity decreased. Precision and F1 score values were low for all models, but were notably higher for models of outbreak month than for models of start month. Models with higher sensitivity also had higher precision and F1 score values.

Using the random forest approach to classify the start month, total rainfall from the previous month (total rain(lag1)), the number of travellers arriving from other PICs (regional arrivals) and the number of travellers from outside the PICs (global arrivals) were selected into the model (Table 3). Regional arrivals was included through forward selection despite a very low variable importance score (Fig 2A). In the model classifying the months with outbreaks, total solar radiation for the current month (total radn) emerged as an additional relevant factor, despite minimum temperature and relative humidity variables having higher importance scores (Fig 2B). Using XGBoost, only two variables were included in the forward selection process for both start month and outbreak months. For start month, the global arrivals variable was the additional variable selected.

## Discussion

Insights into important variables for identifying dengue outbreaks in PICs were gained by comparing and contrasting random forest and XGBoost models and through the use of a forward selection process. The highest balanced accuracy across all models and targets was achieved using forward selection and random forest modelling for the start month of an outbreak (64.2%). This was the only forward selection scenario that resulted in an increase in balanced accuracy, and a larger increase in sensitivity than decrease in specificity compared with the full models. Although model classification skill (balanced accuracy) was too low for the models to be used in a predictive sense, the results from variable importance investigations, and in particular the forward selection process, can offer insights for future research to build upon. The novel use of a forward selection algorithm simplified the model by reducing the number of explanatory variables. Both global and regional arrivals within the target month were included by forward selection in describing start months, despite the difference in their respective importance scores (2). For the random forest models, including regional arrivals may have helped to explain a smaller subset of outbreaks that were otherwise ignored.

For the random forest approach without forward selection, mean balanced accuracy was higher when all variables were used compared to only using meteorological variables (Table 2). However, using XGBoost, the highest balanced accuracy was achieved when only including meteorological variables. Despite this difference between approaches, there were similarities in both relative variable importance and selected features. Under both modelling approaches, human mobility data were considered more important for the start month than for classifying outbreak months in general. Similarly, during forward selection, both random forest and XGBoost included the global arrivals variable for the start month but not for outbreak months. These results highlight the importance of the selection of variables and sugggest the potential impact of travel between PICs and from further abroad on dengue outbreaks warrants further investigation.

In general, random forests have been shown to be competitive with other modelling techniques [26,29]. They also offer an easy analysis of variable importance and may capture non-linear relationships between variables and the target classes. Other modelling techniques could be trialled to explore if more predictive results can be achieved. However, in comparing random forest and XGBoost, both performance and variable importance were similar. Limitations in the data and missing potential drivers of outbreaks such as endemicity may have contributed to the relatively low balanced accuracy scores for all the models explored. These and other limitations need to be addressed in future.

Identifying mass gatherings, as either known international events, or based on changes in arrivals based on flight data, did not contribute to describing outbreak months or start months, regardless of the modelling approach used. While these factors were not considered important at the temporal and spatial scales used in this study, the effect of mass gatherings may have a larger impact at finer spatial or temporal scales.

There are several limitations in the data that need to be considered. First, the key data for human movement, outbreak start and end dates were only available at a monthly timescale. This may not reflect the timescale at which outbreaks evolve. This may have flow-on effects on the importance of mass gatherings, as social events occur on much smaller timescales. Second, data on occurrence and size of the international mass gatherings considered was sparsely available. Third, meteorological data were extracted from Open-Meteo [40] and represent the closest available grid point to capital cities. For the larger PICs, this may not reflect the variability in land-based observations and for small PICs these points may be over oceans, and hence, not accurate. Finally, the nature and definition of outbreaks may have changed over the study period. For example, becoming endemic and therefore less reliant on importation, or being identified as an outbreak when fewer cases are reported.

Two important potential drivers not included in this analysis were endemicity and identification of travel from countries currently experiencing an outbreak. Data on the endemic status of each country is limited, but it is likely that only four PICs could be considered endemic for at least some of the study period. A preliminary importance selection analysis incorporated this PIC endemic state variable but suggested it was not important. This could be due to the small number of PICs classified as endemic during the study period, or the uncertainty regarding which dengue strain was endemic and whether an outbreak is caused by the corresponding serotype. A more detailed analysis is needed to better understand the impact of endemicity and travel from these PICs or other dengue endemic countries on outbreak dynamics. Identifying travel from countries experiencing active outbreaks may provide more informative insights than considering overall travel patterns. However, this approach would require comprehensive global outbreak data, including at least the serotype for each outbreak and, ideally, phylogenetic information to trace the source of specific outbreaks. Including travel from countries currently experiencing an outbreak was beyond the scope of this study, as the outbreak status of non-PIC countries was not known and the serotype of many outbreaks was not known. Removing outbreaks with an unknown serotype may have further weakened model results.

Results from this study suggest several areas for future research. Having data for only a small number of international mass gatherings may have resulted in an underestimation of their importance. Future research should consider how the nature and definition of outbreaks may have changed in the PICs over time, such as changes between endemic and non-endemic transmission conditions, and how this may affect conditions that drive outbreak occurrences. Our study was based on the best available for the time period and region, however, data collection efforts have increased and future research should make use of this improving data set. The connection between dengue outbreaks in PICs and increased travel into PICs also needs to be further explored to better understand how increased travel affects the risk of outbreaks. This may require information at a higher temporal and spatial resolution. One possibility to further investigate the link between human movement (human mobility and international mass gatherings) and disease outbreaks is to consider mobile or social media data as used by other authors [10,22,47]. Finally, future research should consider alternative modelling techniques to compare predictive performance. Such comparisons could consider a reduced set of parameters based on those identified in the current study.

## Supporting information

S1 Algorithm Forward selection algorithm as applied to random forest and XGBoost models. (PDF)
