# Supplementary material for: Investigating linkages between human movement and meteorological variables on dengue outbreaks in the Pacific Islands
Source: PLoS Negl Trop Dis. 2025 Oct 22;19(10):e0013607. doi: 10.1371/journal.pntd.0013607 (PMC12543170; doi:10.1371/journal.pntd.0013607)
Supplement: S1 Algorithm — (PDF) [file pntd.0013607.s001.pdf]

The supplementary algortihm (S1 Algorithm) describes the forward selection of the random forest and XGBoost models. Forward selection was used to simplify the model and identify which variables complemented each other.

Algorithm S1 Forward selection algorithm, where 'classifier' refers to either 'randomForest' or 'XGBoost'.

```
Set current_variables ← best_explanatory_variable
Set current_model ← classifier(y ∼ current_variables)
Set variables ← list_of_variables_other_than_best
Set tol ← 0.01
while do {length(variables) > 0}
  for do {i in 1:length(variables)}
    Record skill and explanatory variable from: classifier(y ∼ current_variables + variables_i)
  end for
  Set candidate_model ← classifier(y ∼ current_variables + best_additional_explanatory_variable)
  Set candidate_model_skill ← skill of classifier(y ∼ current_variables + best_additional_explanatory_variable)
  Set improvement ← skill_candidate_model − skill_current_model
  if then {improvement > tol}
    Set current_variables ← current_variables + best_additional_variable
    Set variables ← variables − best_additional_variable
    Set current_model ← classifier(y ∼ current_variables)
  else
    return(current_model)
  end if
end while
```
